# An Energy Reward-Based Caching Mechanism for Information-Centric Internet of Things

**DOI:** 10.3390/s22030743

**Published:** 2022-01-19

**Authors:** Ngocthanh Dinh, Younghan Kim

**Affiliations:** School of Electronic Engineering, Soongsil University, Seoul 06978, Korea; younghak@ssu.ac.kr

**Keywords:** energy efficiency, Internet of Things, wireless sensor networks, caching energy reward, content store, information objects, J0101

## Abstract

Existing information-centric networking (ICN) designs for Internet of Things (IoT) mostly make caching decisions based on probability or content popularity. From the energy-efficient perspective, those strategies may not always be energy efficient in resource-constrained IoT because without considering the energy reward of caching decisions, inappropriate routers and content objects may be selected for caching, which may lead to negative energy rewards. In this paper, we analyze the energy consumption of content caching and content retrieval in resource-constrained IoT and calculate caching energy reward as a key metric to measure the energy efficiency of a caching decision. We then propose an efficient cache placement and cache replacement mechanism based on the caching energy reward to improve the energy efficiency of caching decisions. Through analysis and experimental results, we show that the proposed mechanism achieves a significant improvement in terms of energy efficiency, stretch ratio, and cache hit ratio compared to state-of-the-art caching schemes.

## 1. Introduction

Information-centric Networking (ICN) is recognized in the literature as one of the most potential networking architectures for the Internet of Things (IoT). ICN takes into account content object and content name at the network level while IoT network traffic is driven mostly by content retrieval, instead of point-to-point communication. With low power devices, resource-constrained IoT networks like wireless sensor networks (WSNs) normally operate using different standards such as 802.15.4, compared to the Internet and normal wireless devices. IoT devices are capable of sensing, collaborating, and interchanging content between them and the Internet. Sharing an IoT infrastructure for multiple applications is a trend nowadays and sharing IoT data to respond to concurrent application requests is energy efficient. As the complicated architecture of the IP model is difficult to handle such interconnection and data exchanging, studies have shown that the re-modeling from end-to-end communication model toward information-oriented model places ICN to meet the requirements of IoT. As a result, operations of the information-centric approach may enhance content access, content distribution, reduce the content retrieval delay, and improve the network performance for IoT.

We note that the pattern of IoT applications follows a content-oriented fashion in which actuators and sensors may not communicate with particular things. IoT applications may demand content based on various properties such as query-based content, event data traffic, monitoring, or critical content. ICN provides opportunities to implement IoT applications in a native view. In [1,2,3], the authors implemented ICN for smart city applications. In [4], the authors implemented ICN for smart home applications using hierarchical names and a multi-party forwarding mechanism for content retrieval from multiple producers. In [5], the authors discussed how ICN can be leveraged for industrial automation. In [6], Boul et al. designed a secured and reliable intelligent transport system using ICN.

In-network caching is the core feature of ICN which places cached content objects around the network and makes content available for requests. The utilization of in-network caching in ICN can improve content availability, so in-network caching is highly beneficial for resource-constrained devices. In ICN in-network caching, a critical issue is to determine which content objects should be cached and which routers should cache a content object. ICN caching schemes for IoT can be categorized into the following groups, graph-based caching, label-based caching, probabilistic caching, and popularity-based caching [7,8,9]. Among those groups, the literature shows that popularity-based caching is one of the most efficient caching approaches for IoT [10]. In popularity-based caching, nodes decide which content objects should be cached based on content access frequency and interest message distribution. This approach is to increase the usage of popular content objects to increase the cache hit ratio. In [11], Precache is an ICN caching scheme based on content relevance. In MPC [12], the authors implemented a way to calculate the content popularity based on counting the number of incoming interest requests for content objects. MPC uses a threshold to determine the popularity level of content objects. When the number of interest requests for a content object is greater than or equal to a threshold, the content object is labeled as popular content. MPC recommends nodes holding popular content objects to cache the content objects. In [13], the authors proposed CPCCS caching scheme which uses a dynamic threshold for least popular content (LPC) and optimal popular content (OPC) and recommend routers along the routing path should cache OPC content objects while fewer routers should cache LPC content objects.

However, the caching schemes above may not always be energy efficient for resource-constrained IoT devices like sensors and actuators where energy efficiency is one of the most concerning factors. The reason is that existing ICN designs for IoT mostly make caching decisions based on probability or content popularity. We admit that those caching strategies are beneficial in many ways. However, considering resource-constrained IoT, those caching strategies may not always be energy efficient when inappropriate routers are selected to cache content objects or inappropriate content objects are selected to be cached. From the energy-efficient perspective, if a node *i* with a low residual energy level $e_{i}$ is selected to cache popular content objects, the node has to serve more content requests than other nodes. Therefore, its energy can be exhausted quickly. This case may not occur in normal cases of the Internet or edge computing because nodes are normally charged. However, with resource-constrained IoT, when the node is out of battery, its cached content objects are not accessible. This aspect can make a caching decision based on probability or popularity without considering energy factors become inefficient in terms of energy when selecting inappropriate content routers. In addition, caching a content object without considering its energy reward can also lead to energy inefficient caching decisions. In many cases, energy consumption to cache and retrieve a cached content object $c_{k}$ from a content router *r* can be even higher than energy consumption to forward interest messages to the original content producer *o*. As explained above, this issue may be not very important in normal cases of the Internet or edge computing because energy consumption may not be the most important factor, but the network performance. However, in resource-constrained IoT like wireless sensors and actuator networks, energy efficiency is considered as one of the most critical factors.

The motivation of this paper is to design an efficient ICN caching mechanism for resource-constrained IoT taking energy efficiency as the key factor. We analyze the energy consumption of caching operations as well as content retrieval in resource-constrained IoT and calculate caching energy reward as the main metric to measure the energy efficiency of a caching decision. We then propose an energy reward-based caching (ERC) mechanism to enhance cache placement and cache replacement in resource-constrained IoT. Our contribution in this paper is three folded. First, we analyze energy consumption for caching and content retrieval in resource-constrained IoT. Second, we propose an energy-efficient cache placement and cache replacement based on caching energy reward. Third, we extensively evaluate the proposed mechanism through analysis and simulation. Through analysis and experimental results, we show that the proposed mechanism achieves a significant improvement in terms of energy efficiency compared to state-of-the-art caching schemes.

The rest of this paper is organized as follows. Section 2 discusses related works. Section 3 gives the overview and the detailed design of the proposed mechanism. Section 4 describes our analysis, experiments, and obtained results. Finally, Section 5 concludes the paper.

## 2. Related Work

In-network caching is one of the main features of ICN to reduce the network load, increase content availability, and lower data delivery latency by allocating cached content objects inside the network and making them available for content requests [10]. If content objects are only available at the content producer or cached nearby the producer, the network around the producer may witness a heavy traffic load and the content delivery latency may be high. If a content object is cached to nearby consumers, requests for the content object can be retrieved faster. One of the critical issues of in-network caching is which routers should cache a content object. Caching schemes in the literature of ICN can be grouped into the following categories, popularity-based caching, probabilistic caching, label-based caching, and graph-based caching which are thoroughly reviewed in the literature [7,8,9].

In probabilistic caching, routers use a probability *p* to make a caching decision. *p* can be a fixed or random number. Probabilistic caching introduces a certain probability of caching for a content object that a router receives. For a given *p*-value, when an ICN router receives a new content object, the router randomly generates a number between 0 and 1. If the generated value is lower than *p*, the router makes a caching decision to cache the content object. Otherwise, the router discards the content object. In [14], the authors solved the issue of unpredictability and simplicity by introducing globally random caching. LCE [15] is a popular and simple version of probabilistic caching but results in high redundancy. The idea behind LCE is very simple in which ICN routers try to cache every new content objects they receive and not available in their CS. In [16,17,18], the authors proposed dynamic caching policies in which the caching probability is changed dynamically to optimize the cache efficiency. HCP [17,18] was implemented using a factor, namely, $CacheWeight_{y}$, to lower the number of similar content replications and another factor, namely $CacheWeight_{M}RT$ to optimize the stretch length between content consumers and content providers.

Label-based caching uses policies related to content objects which are labeled based on certain properties. As a result, nodes may be aware of several content types in the network and have special policies to cache content objects belonging to those content types. In [19,20], the authors take content traffic patterns and the network topology into consideration to design caching schemes that can recognize the network context to improve the network performance.

Graph-based caching considers forwarding routes and network structure in ICN. In [19], the authors proposed an edge caching policy to place content objects in the delivery path end to distribute the content courses close to users to improve the network performance. In [20], the authors discuss various policies to progressively change the caching positions and centrality of nodes.

In popularity-based caching, routers make the caching decision based on the content frequency and interest request distribution. This approach aims at maximizing the usage of popular content to increase the cache hit rate. In Most Popular Cache (MPC) [12], routers count the number of incoming interest messages for every content object to calculate the content popularity. A threshold is defined to categorize content objects as popular. When a content receives a proper number of interest messages greater than the threshold, it is labeled as popular. Routers that hold the popular content are recommended to cache the content. In CPCCS [13], the dynamic threshold value is introduced. The content is grouped into optimal popular content (OPC) and least popular content (LPC). The grouping decision is based on counting the total number of interest messages of a particular content name using PIT. The list of LPC content names is sorted and 25% of total contents from the list are labeled as OPC that are most frequently requested. OPC content objects are recommended to be cached by all routers along the routing path to increase the network performance while LPC content objects are recommended to be cached by fewer routers. In popularity-based caching, collaborations among routers can result in a higher efficiency where routers cooperate to make caching decisions. A number of studies investigated collaborative caching in ICN [21,22,23,24]. The key benefit of collaborative caching strategies is that the redundancy in caching can be lowered and cache diversity can be improved. However, existing collaborative caching strategies have critical drawbacks due to higher communication overhead and packet delivery latency for signaling messages to be exchanged among routers and coordination mechanisms among routers.

Above caching schemes may not always be energy efficient for resource-constrained IoT devices [25,26] like sensors and actuators where energy efficiency is one of the most concerning factors. From an energy-efficient perspective, the reason is that without considering the energy reward of caching decisions, inappropriate routers and content objects may be selected for caching, which may lead to negative energy rewards. In addition, if a node *i* with a low residual energy level $e_{i}$ is selected to cache popular content objects, the node has to serve more content requests than other nodes. Therefore, its energy can be exhausted quickly. This case may not occur in normal cases of the Internet or edge computing because nodes are normally charged. However, with resource-constrained sensors, when the node is out of battery, its cached content objects are not accessible. This paper carefully analyzes the energy reward of caching decisions to increase the energy efficiency of caching in resource-constrained IoT.

## 3. Proposed Energy Reward-Based Caching Mechanism

### 3.1. Caching Energy Consumption Analysis for Resource-Constrained IoT

Table 1 summarizes the acronyms used in this paper. In information-centric IoT, the total energy consumption consists of two main factors, caching energy consumption and transport energy consumption. Transport energy consumption includes energy consumption to forward and process interest messages and content objects from a node to another node. Caching energy of nodes is consumed primarily by content storage at intermediate routers. Caching of content objects in information-centric IoT also raises energy-efficient issues because nodes are resource-constrained devices with a limited residual energy capacity. However, this factor has not been studied thoroughly in ICN IoT. To optimize energy consumption by ICN in IoT, we analyze caching energy consumption as follows.

We denote G=(V,L) as a network graph with $V=v_{1},v_{2},v_{3},\ldots ,v_{N}$ represents a set of N resource-constrained IoT nodes, and *L* represents a set of links among the nodes. We denote *K* as a set of content objects with size $s_{k}$, k=1,2,3,…,K. We assume node *i* in *G* having a cache size of $c_{i}$ and residual energy $e_{i}$. Interest messages for content objects are sent with a rate $\lambda =\sum_{k=1}^{K}\lambda_{k}$, where $\lambda_{k}$ is the request rate, in other words, the content access frequency of *k*. We use $X_{i,k}$ as a boolean value of 0 or 1 to indicate whether the content object *k* is cached at node *i*. We denote $H_{i,k}$ as the number of hops that the interest message traveled to retrieve *k*, $P_{ca}$ as energy consumption rate to cache 1 bit of content in a unit of time, $P_{L}$ as energy consumption rate to transmit 1 bit of content, and $P_{t}$ as energy consumption rate for a node to check and forward 1 bit of content. We calculate caching energy consumption $E_{ca}$ at intermediate nodes in a time interval *t* as follows.

(1) $$E_{ca}=\sum_{i=1}^{N}\sum_{k=1}^{K}E_{ca}^{i,k}=\sum_{i=1}^{N}\sum_{k=1}^{K}P_{ca}tX_{i,k}s_{k}$$

We assume that the energy consumption rate to process a message of interest is equal to that of a content object. We then calculate transport energy consumption comprising energy consumption for packet processing and link energy consumption, as follows.

(2) $$E_{tr}=\sum_{i=1}^{N}\sum_{k=1}^{K}E_{tr}^{i,k}=\sum_{i=1}^{N}\sum_{k=1}^{K}\left[P_{L}H_{i,k}+\left(2H_{i,k}-1\right)P_{t}\right]s_{k}\lambda_{i,k}$$

We denote *r* as a content router caching a content object $c_{k}$. The consumer of $c_{k}$ is M hops from *r* and Q hops from the content producer *o* of $c_{k}$. Now we consider the time interval *t* as the average interval between two interest messages for $c_{k}$ at *r* into (1). The caching energy reward of *r* to cache $c_{k}$, $E_{r}^{k^{reward}}$ is calculated as follows.

(3) $$E_{r}^{k^{reward}}=E_{tr}^{o,k}-\left[E_{ca}^{r,k}+E_{tr}^{r,k}\right]$$

The caching energy reward of *r* to cache $c_{k}$, $E_{r}^{k^{reward}}$ is positive only if $E_{r}^{k^{reward}}=E_{tr}^{o,k}-\left[E_{ca}^{r,k}+E_{tr}^{r,k}\right]>0$. It means that energy consumption to retrieve content object *k* from the content router *r* and energy consumption to cache *k* at *r* should be smaller than energy consumption to retrieve content object *k* directly from its content producer *o*. Our analysis shows that the caching energy reward for content $c_{k}$ not only depends on the popularity of $c_{k}$, measured by the number of interest messages sent for $c_{k}$, but also the content access frequency, measured by the number of interest messages sent for $c_{k}$ in a unit of time.

Existing ICN designs for resource-constrained IoT mostly make caching decisions based on probability or content popularity. We admit that those caching strategies are beneficial in many ways. However, considering resource-constrained IoT nodes, those caching strategies may not always be energy efficient when inappropriate routers are selected to cache content objects or inappropriate content objects are selected to be cached. From an energy-efficient perspective, if a node *i* with a lower energy level $e_{i}$ is selected to cache popular content objects, the node has to serve more content requests than other nodes. Therefore, its energy can be exhausted quickly. This case may not occur in normal cases of the Internet or edge computing because nodes are normally charged. However, with resource-constrained nodes, when the node is out of battery, its cached content objects are not accessible. This aspect makes a caching decision based on probability or popularity without considering energy factors become inefficient in terms of energy when selecting inappropriate content routers to cache. In addition, caching a content object without considering its energy reward can also lead to energy inefficient caching decisions. In many cases, energy consumption to cache and retrieve a cached content object *k* from a content router *r*, $E_{ca}^{r,k}+E_{tr}^{r,k}$, can be even higher than energy consumption to forward requests to the original content producer *o*, $E_{tr}^{o,k}$. Therefore, appropriate schemes have to be designed to address these issues.

### 3.2. Energy Reward-Based Caching Mechanism

**Figure 1:**
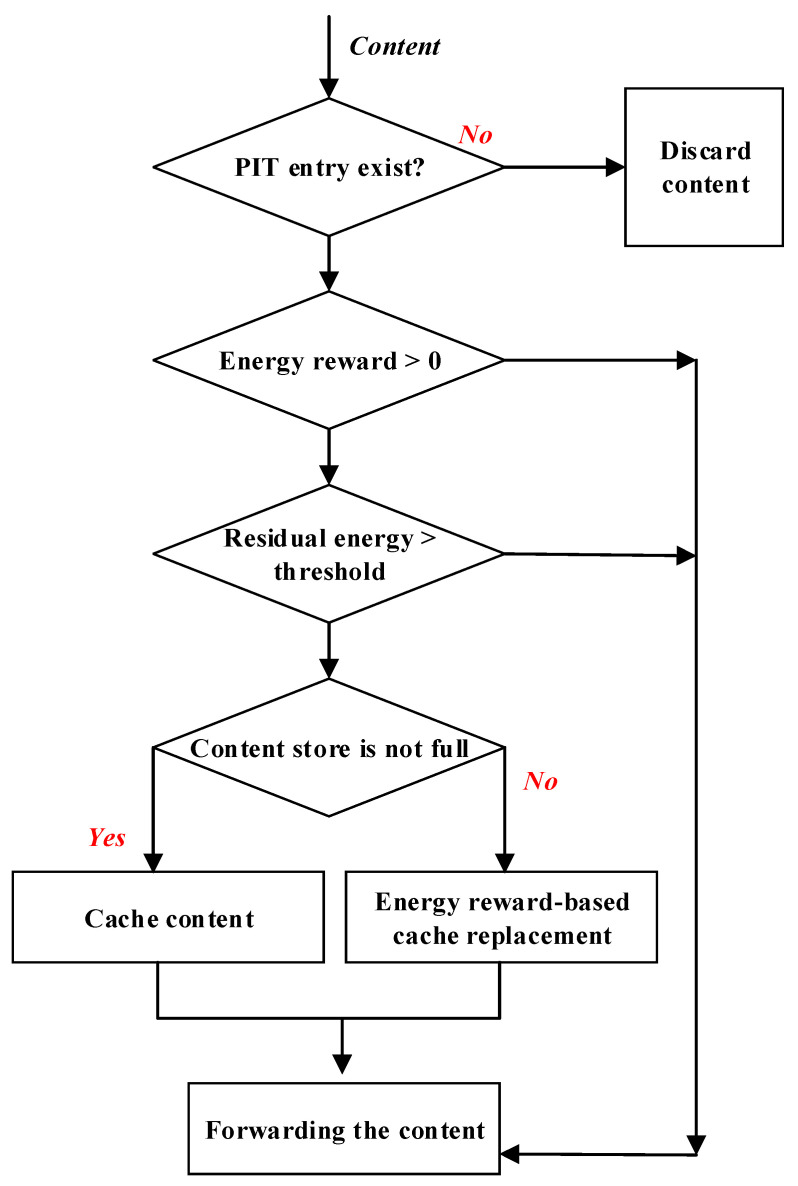
The processing of content objects in the proposed ERC mechanism.

Operations of the proposed energy reward-based caching (ECR) mechanism are illustrated in Figure 1. When a node *r* receives a content object $c_{k}$, the node calculates the energy reward for caching $c_{k}$, $E_{r}^{k^{reward}}$. If the energy reward is positive, the node checks its residual energy. If its residual energy is greater than or equal to an energy threshold $E_{\left|r\right|}$, the node decides to cache $c_{k}$. If its storage is full, the node replaces an existing content object $c_{h}$ in its CS with $c_{k}$ if the energy reward to cache $c_{k}$, $E_{r}^{k^{reward}}$, is higher than the energy reward to cache $c_{h}$, $E_{r}^{h^{reward}}$. It means that caching $c_{k}$ is more energy beneficial than caching $c_{h}$. If the energy reward is smaller than or equal to 0, the node decides not to cache $c_{k}$ because caching $c_{k}$ doesn’t help improve the energy efficiency of the network. If the residual energy of the node is low, it decides not to cache $c_{k}$, another node in the forwarding path with enough residual energy may cache $c_{k}$ instead of the node.

According to (3), the transport energy consumption to the original content producer $E_{tr}^{o,k}$ is fixed for a content object $c_{k}$. As a result, firstly, the caching energy reward depends on the transport energy consumption from the consumer to the content router $E_{tr}^{r,k}$. Following (2), the proposed cache placement and cache replacement mechanism tend to explore nodes nearby consumers as content routers of $c_{k}$ to minimize $H_{i,k}$, thus increasing the caching energy reward. Secondly, the caching energy reward depends on the caching energy consumption, $E_{ca}^{r,k}$, at the content router *r*. Because $E_{ca}^{r,k}$ depends on the average interval between two interest messages for $c_{k}$ at *r*, the proposed mechanism tends to cache content objects having a high content access frequency for a lower value of *t* to increase the caching energy reward.

Considering the energy efficiency perspective, the proposed mechanism makes a caching decision for only contains objects that create energy-saving benefits for the network. It implicitly indicates that a content object should have a content access frequency high enough so that energy saving by reducing transport energy consumption to forward interest messages for the content object to the original content producer is higher than the energy consumption of the content router to cache the content object. Note that, this work focuses purely on energy efficiency aspects of caching in information-centric IoT. In practice, the network performance (i.e., content retrieval latency) is also an important metric. Therefore, there are cases that even though energy reward $E_{r}^{k^{reward}}$ is lower than or equal to 0, making a caching decision still returns benefits in terms of the network performance. For example, in the case of a content delivery network (CDN) which is implemented to mainly optimize the network performance. However, considering resource-constrained IoT, the idea behind the proposed mechanism is to optimize mainly for energy efficiency.

## 4. Performance Evaluation

We implement ERC and conduct simulations using the COOJA simulator in Contiki [27] with 1050 nodes in a coverage area of 1000 m × 1000 m. Nodes are deployed randomly with sensing correlation obtained from the sensor data collected from the real-world IntelLab deployment [28]. The system generates content requests from random nodes following Zipf-like distribution. The CS storage capacity of each cache node is varied from 5 to 25 content objects. As implemented in our prior study [29], we reuse HTTP-CoAP converter in this paper to convert application requests of consumers in HTTP to CoAP for IoT nodes. Application requests are encoded using templates in extensible markup language (XML) and decoded using SensorML interpreter for IoT nodes [30]. For data collection, we utilize CTP and LPL [29] as the data collection schemes and 802.15.4 MAC (Media Access Control) mechanism. We use closest-fit-pattern matching (CPM) as the radio noise model [29]. We use CCA (clear channel assessment) [29] check parameter up to 400 times. We set the residual energy threshold $E_{\left|r\right|}$ for each node is 10%.The detailed parameter configurations of simulations are shown in Table 2. Other parameters remain as same as the default configurations of the Contiki CC2420 radio model [29]. By default, we use the cache size of 20 content objects and a wakeup interval of 1 s if those parameters are not specified. The naming scheme [31] is used for IoT nodes. The experimental results are reported at 96% confidence interval. Through analysis and experiments, We show the performance evaluation of ERC in comparison with state-of-the-art caching schemes MPC [12] and CPCCS [13].

We use the following metrics for the performance evaluation and comparison.

Average radio duty cycle: We use average radio duty cycle as an indicator for energy efficiency [32]. We consider timing aspects for calculating the duty cycle (e.g., time for transmission). A radio duty cycle of a node is the ratio of the radio active period and the cycle time, the cycle time is the duration of active time and the sleep time of IoT nodes. The overall duty cycle (DC) of a node *i* is calculated using (1) by simply adding duty cycles for each radio operation: listening ($DC_{lx}$), transmitting ($DC_{tx}$), receiving ($DC_{rx}$), overhearing ($DC_{over}$), and additional operations ($DC_{add}$) [32].

(4) $$DC_{i}=DC_{i}^{lx}+DC_{i}^{tx}+DC_{i}^{rx}+DC_{i}^{over}+DC_{i}^{add}$$

To measure the radio duty cycle, we record changes in the radio’s states and use a counter to accumulate the time period used in each state. At the end of the simulation, we calculate the average radio duty cycle and report average results.

The average duty cycle of nodes in a network is calculated as follows.

(5) $$DC_{average}=\frac{\sum_{i=1}^{n}DC_{i}}{n}$$

where *n* is the total number of IoT nodes.

Average stretch ratio (ST): the hop distance forwarded of a message of interest from the content consumer toward a content provider is known as stretch. We calculate the stretch ratio as follows.

(6) $$ST_{average}=\frac{\sum_{i=1}^{I}\frac{H_{i}^{forwarded}}{H_{i}^{c-p}}}{I}$$

where *I* is the total number of interest messages sent to the network, $H_{i}^{forwarded}$ is the number of hops that the interest message *i* is forwarded until satisfied, $H_{i}^{c-p}$ is the total number of hops from the consumer to the content producer of interest message *i*.

**Figure 2:**
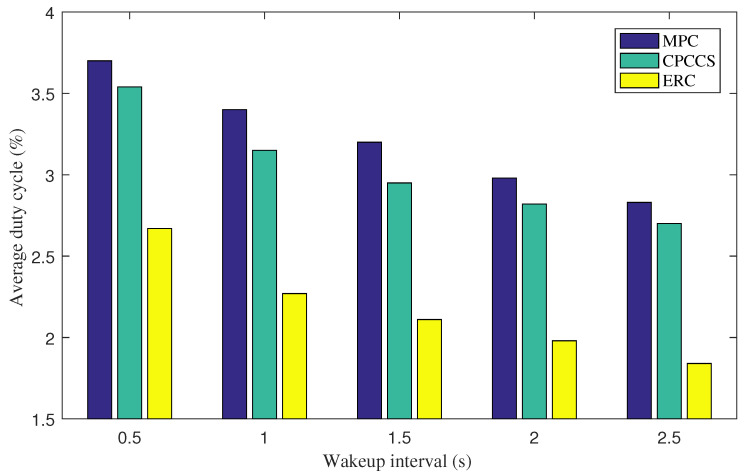
Average radio duty cycle under various wakeup intervals.

Figure 2 shows the average radio duty cycle of nodes under various values of wakeup interval. The proposed mechanism achieves a lower average duty cycle compared to MCP and CPCCS. This indicates that the proposed mechanism helps improve the energy efficiency of nodes significantly compared to MCP and CPCCS. The results are due to our efficient design to select content routers and content objects for caching by carefully considering whether caching a content object at a content router is beneficial in terms of energy or not. In particular, average duty cycle values of nodes at wakeup intervals of 1.5 s of MCP, CPCCS, and ERC are 3.2%, 2.95%, and 2.11%, respectively. Overall, ERC achieves an energy-efficient improvement ratio of over 25%. When we increase the wakeup interval from 0.1 s to 2.5 s, the average duty cycle of nodes decreases. This is due to the fact that when the wakeup interval increases, nodes wake up less regularly.

**Figure 3:**
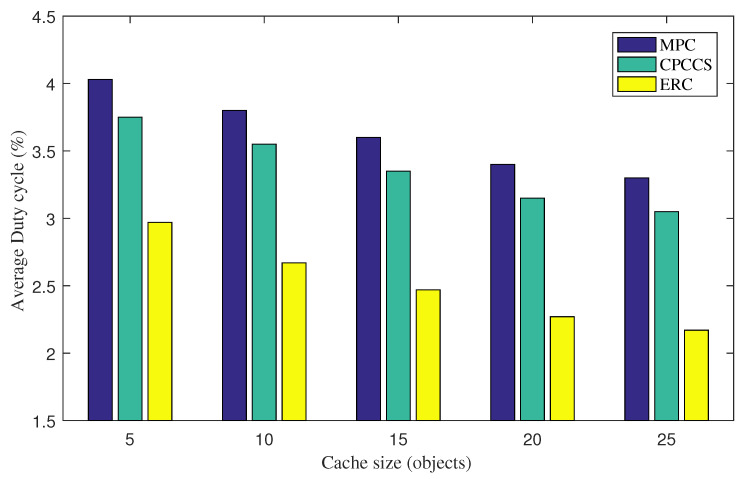
Average radio duty cycle under various cache sizes.

We now fix the wakeup interval of 1 s and increase the cache size from 5 to 25 to study behaviors of the proposed mechanism under changing network conditions. Figure 3 presents the average radio duty cycle of nodes under various cache sizes. Nodes running ERC witness the lowest radio activity for saving energy, compared to MCP and CPCCS. Average duty cycle values of MPC, CPCCS, and ERC at the cache size value of 10 are 3.8%, 3.55%, 2.67%, respectively. We find that the greater the cache size is used, the better the energy efficiency the three caching schemes achieve. The reason is that with greater cache size, nodes can cache a higher number of content objects to reduce forwarding activities.

**Figure 4:**
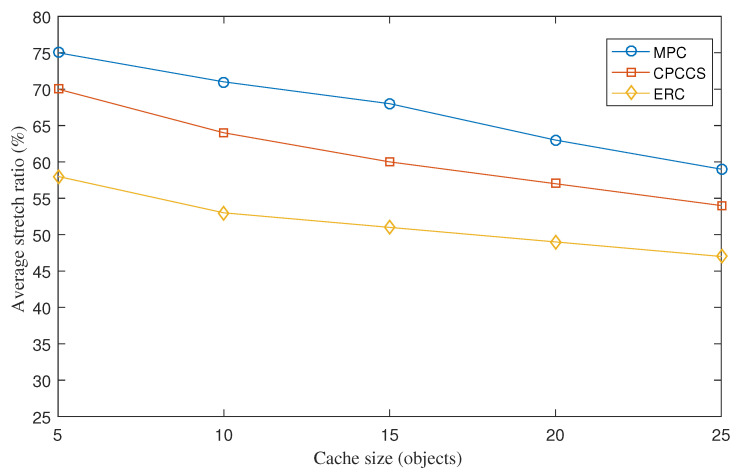
Average stretch ratio under various cache sizes.

The average stretch ratio measures the ratio between the following two metrics: (1) the hop distance that a message of interest is forwarded from the content consumer to the content provider that can be any router on the forwarding path and (2) the hop distance from the content consumer to the content producer. Figure 4 depicts the stretch ratios of MPC, CPCCS, and ERC under various cache sizes. The results presented in Figure 4 can partially help explain the reasons behind the results shown in Figure 2 and Figure 3. This Figure shows that ERC achieves a significantly lower number of hops required to forward interest packets and content objects from and to content consumers. This is due to the design that the proposed cache placement and cache replacement mechanism tend to explore nodes nearby consumers as content routers to minimize the number of hops to increase the caching energy reward. Moreover, the proposed mechanism also tends to cache content objects having a high content access frequency for a lower value of time interval *t* between interest messages to increase the caching energy reward. According to the analysis in Section 3.1, transport energy consumption is proportional to the number of hops, $H_{i,k}$ that the interest message traveled to retrieve content object *k*. By lowering the average number of hops, ERC helps lower the overall energy consumption. When we increase the cache size, the average stretch ratio decreases because more and more content objects can be cached and retrieved at a short distance from intermediate content routers.

**Figure 5:**
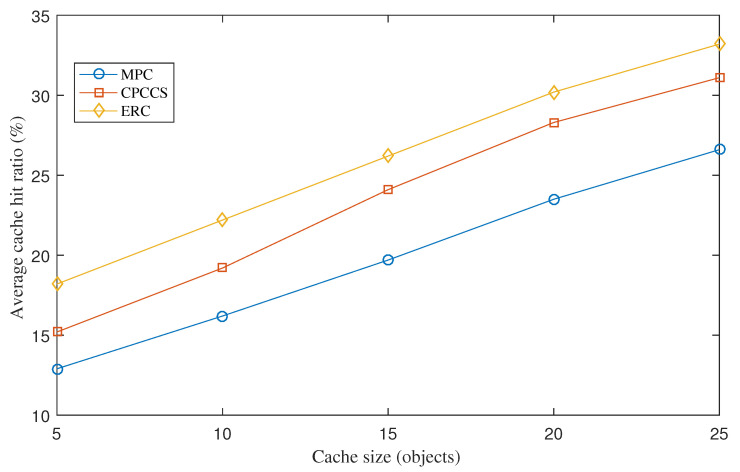
Average cache hit ratio under various cache sizes.

Figure 5 shows average cache hit ratios of MPC, CPCCS, and ERC under various cache sizes. Cache hit ratio results of the three mechanisms increase gradually when we increase the cache size. The Figure shows that ERC witnesses the highest cache hit ratio. A result of interest is that the lower the cache size is set, the higher the improvement ratio ERC achieves. The reason is that ERC exploits limited cache capacity to store content objects that have more access frequency and explores nodes nearby consumers as content routers to optimize the caching reward. When we increase the cache size of nodes, nodes in all three cases are able to cache more content objects which can be popular or less popular. Therefore, the improvement ratio of ERC is lower. This implicates that ERC is more efficient for resource-constrained IoT nodes.

## 5. Discussion and Conclusions

The main motivation behind this work is to propose an energy-efficient caching mechanism for information Internet of Things by considering energy efficiency as the key factor. We conduct extensive analysis for energy consumption of caching operations and packet forwarding to compute caching energy reward for caching decisions. Based on caching energy reward, we design an energy reward-based caching (ERC) mechanism to enhance cache placement and cache replacement in IoT. Through analysis and experimental results, we show that the proposed mechanism achieves a significant improvement in terms of energy efficiency, stretch ratio, and cache hit ratio compared to state-of-the-art caching schemes. The limitation of this work is that we focus purely on energy-efficient factors. In future works, we plan to find an approach to combine energy reward with other metrics to design a mechanism that optimizes multiple objectives for the Internet of Things.

## Figures and Tables

**Table 1:** List of Acronyms.

Acronym	Meaning
ICN	information-centric networking
CS	content store
CDN	content delivery networks
PIT	pending interest table
IoT	Internet of Things
ERC	energy reward-based caching
CPM	closest-fit patterm
NDN	named data networking
CCN	content centric networking
CO	content object

**Table 2:** Parameters.

Parameter	Value	Parameter	Value
Number of nodes	1050	CCA check	400 times
Module current draw	1.8 mA	cache size p	5–25 objects
RF transceiver current draw	23 mA	MAC	LPL
Wakeup interval	0.5–2.5 s	Memory standby draw	50 µA
Transmission current consumption	20.3 mA	Receiving current consumption	18.75 mA
Processing current consumption	2.21 mA	Sleeping mode current consumption	0.67 mA
RF power	−24 dBm to 0 dBm	noise model	CPM
